# Delving into the Role of α-Smooth Muscle Actin as a Predictive Biomarker of Early Recurrence in Invasive Ductal Adenocarcinoma of Breast

**DOI:** 10.30699/ijp.2024.2022442.3255

**Published:** 2024-07-15

**Authors:** Seetu Palo

**Affiliations:** Department of Pathology and Laboratory Medicine, All India Institute of Medical Sciences, Bibinagar, Telangana, India

Dear Editor, 

I found the recent publication by Andrianto *et al.* (1) in the Iranian Journal of Pathology, titled "α-Smooth Muscle Actin as Predictors of Early Recurrence in Early-Stage Ductal Type Breast Cancer After Mastectomy and Chemotherapy," intriguing. This study, employing a cross-sectional immunohistochemical approach, investigates the significance of α-smooth muscle actin (α-SMA) as a potential biomarker for early recurrence in invasive ductal adenocarcinoma (IDC) of the breast. In the current era of precision medicine, it is apt to be on the look for novel biomarkers in predictive oncology, especially in breast cancer which remains one of the leading causes of cancer-related mortality in women despite scientific advancements. However, it's crucial to thoroughly understand the scientific methodology to fully grasp the paper's findings and utilize the same in clinical practice or research settings.

In the context of breast cancer, α-SMA is generally expressed by a diverse population of stromal cells, including native myoepithelial cells, cancer-associated fibroblasts (CAFs), myofibroblasts, blood vessels, and recently many investigators have shown α-SMA to be a poor prognostic biomarker (2-4). Although preliminary results suggest that epithelial tumor cells of breast cancer can also potentially express α-SMA, it remains ambiguous due to paucity of literature in this regard (4). The authors have mentioned that immunohistochemical assessment of α-SMA was performed by assessing the staining intensity and proportion of positive tumor cells (1). It is unclear whether only the tumor cells were taken into account, or the α-SMA positive stromal cells were also included for computing the H-score. Moreover, representative histopathological and immuno-histochemical microphotographs are not available for readers to refer to and infer. Similarly, a few other clinicopathological parameters addressed in the article need to be defined further for better comprehension, namely: (i) Mean (and range) interval of surgery to local recurrence in months (instead of ‘within two years’) ; (ii) Mean (and range) tumor size (instead of pT1 and pT2) in both the groups to ensure homogeneity; (iii) Mean (and range) distance of the invasive tumor front from the closest surgical margin (instead of just ‘tumor free’); (iv) Follow-up data in detail, especially for patients in ‘without recurrence’ group as loco-regional recurrence can occur at varying time intervals (5). Interestingly, the authors observed high α-SMA expression in six cases in the ‘without recurrence’ group, which amounts to almost 1/4^th^ (n=6/25; 24%) of the cohort size. Although the authors have performed bivariate and multivariate analyses encompassing various clinicopathological parameters and levels of α-SMA expression of the whole study sample, a sub-group analysis of these six cases might bring out some important facts relevant to the study’s objective.

It is widely recognized that the status of ER, PR, Her2, and Ki-67 are critical for predicting outcomes and recurrence in breast cancer patients (5-7). Other significant predictive biomarkers for loco-regional recurrence include lympho-vascular invasion, lymph node ratio (number of positive lymph nodes/ number of lymph nodes dissected out), and tumor multifocality (6,8). These important parameters are not reported or discussed by the authors. I think it is essential to compare and contrast both the study cohorts on these parameters to obtain insights about the tumor biology of both groups and correlate α-SMA expressivity with clinical outcome in light of these confounding factors that might have contributed to tumor recurrence. Nonetheless, I commend the authors for their credible scientific contribution, and I believe these observations could aid in a better interpretation of this study.
